# Drivers of stability and transience in composition-functioning links during serial propagation of litter-decomposing microbial communities

**DOI:** 10.1128/msystems.01220-22

**Published:** 2023-05-03

**Authors:** Eric R. Moore, Dennis Suazo, Joany Babilonia, Kyana N. Montoya, La Verne Gallegos-Graves, Sanna Sevanto, John Dunbar, Michaeline B. N. Albright

**Affiliations:** 1 Bioscience Division, Los Alamos National Laboratory, Los Alamos, New Mexico, USA; 2 Earth and Environmental Sciences Division, Los Alamos National Laboratory, Los Alamos, New Mexico, USA; Pacific Northwest National Laboratory, Richland, Washington, USA

**Keywords:** bacteria, carbon cycling, fungi, microbial interactions, microbiome engineering, serial propagations

## Abstract

Biotic factors that influence the temporal stability of microbial community functioning are an emerging research focus for the control of natural and engineered systems. The discovery of common features within community ensembles that differ in functional stability over time is a starting point to explore biotic factors. We serially propagated a suite of soil microbial communities through five generations of 28-day microcosm incubations to examine microbial community compositional and functional stability during plant litter decomposition. Using dissolved organic carbon (DOC) abundance as a target function, we hypothesized that microbial diversity, compositional stability, and associated changes in interactions would explain the *relative* stability of the ecosystem function between generations. Communities with initially high DOC abundance tended to converge towards a “low DOC” phenotype within two generations, but across all microcosms, functional stability between generations was highly variable. By splitting communities into two cohorts based on their relative DOC functional stability, we found that compositional shifts, diversity, and interaction network complexity were associated with the stability of DOC abundance between generations. Further, our results showed that legacy effects were important in determining compositional and functional outcomes, and we identified taxa associated with high DOC abundance. In the context of litter decomposition, achieving functionally stable communities is required to utilize soil microbiomes to increase DOC abundance and long-term terrestrial DOC sequestration as one solution to reduce atmospheric carbon dioxide concentrations. Identifying factors that stabilize function for a community of interest may improve the success of microbiome engineering applications.

**IMPORTANCE:**

Microbial community functioning can be highly dynamic over time. Identifying and understanding biotic factors that control functional stability is of significant interest for natural and engineered communities alike. Using plant litter–decomposing communities as a model system, this study examined the stability of ecosystem function over time following repeated community transfers. By identifying microbial community features that are associated with stable ecosystem functions, microbial communities can be manipulated in ways that promote the consistency and reliability of the desired function, improving outcomes and increasing the utility of microorganisms.

## INTRODUCTION

With an increasing body of research describing links between microbial composition and ecosystem functioning (1 - 3), there is growing interest in manipulating microbial communities to achieve desired functional outcomes. Examples include altering the microbiome in the human gut for improved health (4), in the plant rhizosphere for increased crop yield (5), and in soils for carbon storage (6, 7). However, the reliable manipulation of microbial communities to achieve predictable and persistent functional outcomes remains a challenge (8, 9). A greater understanding of factors that shape the temporal stability of microbial community composition and functioning in different contexts may improve the success of microbiome engineering. This focus is motivated in part by the idea that variation in community assembly can lead to alternative states that differ in functioning and stability (10, 11).

Alternative stable states have been a recurring, albeit controversial, theme in ecology since the 1960s (12 - 14). In this concept, microbiome composition is not determined exclusively by the environment, and therefore different community states are possible under the same environmental parameters (15 - 18). Alternative stable states can arise in otherwise identical environments following abiotic (e.g., nutrient addition) (19, 20) or biotic perturbations (e.g., addition or removal of predators) (21, 22). Alternative stable microbial community types can account for long-term variation in ecosystem functioning within a single environment; however, identifying factors that underpin the stability of microbial ecosystem functioning may offer valuable strategies for microbiome engineering or improving models of global elemental cycles.

One way to probe the stability of the relationship between microbial community composition and ecosystem functioning over time is through serial propagation of communities in highly controlled environments (i.e., community “evolution” experiments). Experimental evolution with single microbial populations has been repeated with various organisms over thousands of generations (23). These experiments have shown that evolution can either be highly convergent (24 - 26) or divergent (27, 28). Extending to the community level, it is plausible that experimental community evolution, which we define as changes in community composition and function during serial propagation over time, may reveal communities that vary in the stability of composition and functioning (29, 30). While the functioning of communities may inevitably change over time, differences in the rate of change (i.e., higher or lower relative stability) can provide insights into the taxa and/or ecological processes that might be augmented to achieve increased stability. Instead of adaptation occurring via mutation at the gene level as seen in pure-culture studies, community outcomes may be driven by changes in ecological processes such as organism interactions that affect the abundance and activity of individual taxa. This raises two key questions about mechanisms that could affect and help predict the functional stability of the communities: (i) Do the communities exhibit variation in the relative stability of composition or functioning over multiple “generations”? and (ii) Are any microbial traits correlated with the observed degree of stability in ecosystem functioning?

To answer these questions, we conducted a community evolution experiment with 10 unique microbial communities using a plant litter decomposition system. Understanding community factors that are linked to the stability of ecosystem functions in litter decomposer communities is central to manipulating and modeling soil carbon (C) cycling. Plant litter decomposition in terrestrial ecosystems is an inherently cyclic process where new litter is periodically deposited onto the soil surface, creating a natural opportunity for serial propagation of microbial decomposer communities that assembled previously on prior batches of litter. As microbial communities assemble on new litter, they undergo ecological succession as the litter is decomposed (31, 32). During the decomposition process, microbes move C between a number of different pools. Some C is respired as carbon dioxide (CO_2_), some C is incorporated into microbial biomass, and some C is transformed into other organic C compounds (33). Dissolved organic carbon (DOC), comprising plant and microbial residues, is a C pool of particular interest in soil carbon cycling because it has the potential to be transported to deeper soil horizons where stabilization and long-term sequestration can occur (34, 35). Changing DOC fluxes may be a route to boost soil C accumulation (36). Recent work has shown that microbial community composition impacts DOC abundance during litter decomposition (7, 11).

To set up the experiment, we used 10 communities representing the highest and lowest DOC abundance among 53 communities originally screened in a preliminary common garden experiment in sealed microcosms containing blue grama (*Bouteloua gracilis*) grass litter inoculated with microbiomes derived from different soils. To examine community changes following the initial generation, we sequentially propagated four replicates of the 10 down-selected litter decomposer communities for four “generations” (28 days per “generation”) in microcosms containing blue grama litter and sand. The primary metric of community functioning was the concentration of DOC observed after 28 days of litter decomposition, and CO_2_ accumulation was also measured. We hypothesized that microbial diversity, community stability, and associated changes in interactions would explain the relative stability of community functioning. Our findings show that litter-decomposing communities varied widely in their functional stability, and we identified microbial and physiochemical features linked to increased stability in community functioning. In particular, we found that compositional shifts, interactions among diversity and environmental parameters, and interaction network complexity were associated with the stability of DOC abundance between generations. Further, our results showed that legacy effects were important in determining compositional and functional outcomes, and we identified taxa associated with high DOC abundance. Identifying factors that control functional stability will be useful for understanding variability in carbon flow in plant litter–decomposing communities and may increase successful applications of microbiome engineering in soils and other systems to achieve desired outcomes such as increasing soil C sequestration.

## MATERIALS AND METHODS

### Soil collection and microbial community inoculum

To obtain diverse soil microbial communities, we collected 53 soils from seven elevation transects in the southwestern USA (Table S1). These soils were used to create complex microbial community inoculum (20× soil dilution) using the following protocol: 1.0 g of sieved soil was mixed with 9.0-mL deionized H_2_O to make a 1:10 dilution, and the soil suspension was shaken and then allowed to sit for 2 min to allow the largest soil particles to settle. Five milliliters of the supernatant was removed and added to 5.0 mL of 10 mg/mL ammonium nitrate (NH_4_NO_3_) solution to create a 1:20 soil slurry at a final concentration of 5.0 mg/mL NH_4_NO_3_.

### Artificial selection and serial propagation microcosm experiment

To screen the microbiomes for high and low DOC abundance, microcosms were constructed using 125-mL glass serum bottles with one tablespoon of Quikrete play sand (~7 g) and 100 mg of 1-cm cut blue grama (*B. gracilis*) litter. Before use, microcosms were sterilized via autoclave three times (at 121°C and 15 psi) for 1 hour with at least 8 hours between each sterilization. For the initial generation (G_0_), we inoculated 1 mL of each of the 53 different soil slurry inocula obtained from the seven elevation transects (see Table S1 and Fig. S1 in the supplemental material) into duplicate microcosms (*n* = 106). Microcosms were sealed with Teflon-lined crimp caps (preventing desiccation) and incubated in the dark at 25°C for 28 days. CO_2_ was measured by gas chromatography five times over the course of 28-day incubation period using an Agilent Technologies 490 Micro GC (Santa Clara, CA, USA), and these measurements were summed to calculate the total CO_2_ accumulation during the 28-day incubation period. After each measurement, the headspace air was evacuated with a vacuum pump and replaced with sterile-filtered air to prevent CO_2_ buildup.

At the end of the 28-day incubation period, microcosms were destructively sampled. Ten milliliters of sterile distilled water was added to the microcosms, and they were shaken for 30 s to homogenize and create a slurry. An aliquot of the slurry was filtered through a 0.22-μM filter, and DOC and total nitrogen (TN) concentrations were measured on an OI Analytical model 1010 wet oxidation TOC analyzer (Xylem Inc., Rye Brook, NJ, USA). A subset of the G_0_ microcosms were selected to propagate microbial communities based on their DOC concentrations (Fig. S1). Specifically, five source communities with the highest mean DOC (*n* = 2), and five others with the lowest mean DOC were selected for propagation (Fig. S1). Each of these communities were derived from soils taken from different transects, or elevations within a transect (Table S1 and Fig. S1). Variation between duplicates of these 10 selected communities was less than those with intermediate values of DOC abundance (Fig. S1). For the next generation (G_1_), a new set of microcosms containing one tablespoon of (~7 g) sterile sand and 100 mg of 1-cm cut blue grama (*B. gracilis*) litter were inoculated with an unfiltered 1 mL aliquot of slurry (20× dilution final concentration of 5 mg/mL NH_4_NO_3_) from these down-selected G_0_ microcosm communities. Each selected individual G_0_ community replicate was used to inoculate two new microcosms in G_1_, for a total of 40 communities (10 communities from different origins × four replicates). As with G_0_, the 40 G_1_ microcosms were incubated for 28 days at 25°C and then serially propagated through three subsequent 28-day generations (G_2_, G_3_, G_4_) in microcosms using the same setup and conditions (Fig. S1). The entire experiment consisted of a total of five 28-day generations and a total of 140 days of incubation time. As with G_0_, CO_2_ was measured five times over the course of each generation and summed to calculate total CO_2_ accumulation during each generation to obtain a more complete view of the carbon cycle. Samples were also collected at the end of each generation for DOC/TN analysis using the same methods described above for G_0_. Sand, litter, and liquid residue were collected from the microcosms at the end of each generation (G_0_, G_1_, G_2_, G_3_, G_4_) and stored at −20°C until DNA extraction.

### Microbial community taxonomic profiling

For the microcosm samples, we extracted and sequenced DNA to obtain bacterial (16S rRNA) and fungal (internal transcribed spacer [ITS]) community profiles. DNA extractions were performed with a DNeasy PowerSoil Kit 96-well HTP kit (Qiagen, Hilden, Germany) following the manufacturer’s protocol with the following exceptions, 0.3 g of material was used per sample extract and all samples were eluted to a final volume of 30 μL. The DNA samples were quantified with the Invitrogen Quant-iT dsDNA Assay (HS) kit (Invitrogen, Waltham, MA, USA), following the manufacturer’s protocols on BioTek Synergy HI Hybrid Reader. The V4 region of bacterial 16S rRNA gene was amplified with methods previously described by Albright (11). PCR2 amplicons were cleaned with a 0.9 ratio of Beckman Coulter Agentcourt AMPure XP beads (Beckman Coulter, Brea, CA, USA). Following cleanup, samples were quantified using the same method as the extracted DNA and then pooled to 10 ng each. The pool was then cleaned with a 0.9 ratio of Beckman Coulter Agentcourt AMPure XP beads following manufacturer’s protocol. Fungal ITS sequences were amplified using an equimolar mixture of three ITS9 forward primers (ITS9f_FS1: TCGTCGGCAGCGTCAGATGTGTATAAGAGACAGNNNNNNGAACGCAGCRAAIIGYGA, ITS9f_FS2: TCGTCGGCAGCGTCAGATGTGTATAAGAGACAGNNNNNGAACGCAGCRAAIIGYGA, and ITS9F_FS3: TCGTCGGCAGCGTCAGATGTGTATAAGAG­ACAGNNNNGAACGCAGCRAAIIGYGA) and the ITS4r_FS reverse primer (GTCTCGTGGGCTCGGAGATGTGTATAAGAGACAGNNNNNNTCCTCCGCTTATTGATATGC) (37). ITS amplification was set up using Phusion Hot Start II High Fidelity DNA polymerase (Thermo Fisher Scientific, Vilnius, Lithuania). In the first PCR, barcoded amplicons were produced over 25 cycles using gene primers. Cycling conditions were 5 min at 95°C, 25 cycles (95°C for 30 s, 50°C for 60 s, 68°C for 60 s), and a final extension step of 68°C for 10 min. The second PCR extended Illumina adapter sequences on the amplicons over 12 cycles. Cycling conditions were 5 min at 95°C, 12 cycles (95°C for 30 s, 50°C for 60 s, 68°C for 60 s), and a final extension step of 68°C for 10 min. Amplicons were cleaned using 0.9 ratio of Beckman Coulter Agentcourt AMPure XP beads, quantified using the same procedure as for the extracted DNA and then pooled at a concentration of 10 ng each. The pooled samples were further cleaned and concentrated using a 0.9 ratio of Beckman Coulter Agentcourt AMPure XP beads following manufacturer’s protocol. Pooled samples were eluted to a final volume of 30 μL in Qiagen EB Buffer. DNA quality of the amplicon pool was assessed with a bioanalyzer, and concentration was verified by quantitative PCR.

Amplicon libraries were sequenced using an Illumina MiSeq to generate 300-bp (base pair) paired-end reads. Bacterial 16S sequence reads were preprocessed and demultiplexed using USEARCH (38). DADA2 was used with default settings, unless otherwise noted, to perform quality filtering, primer removal, and denoising (39). Quality filtering of bacterial reads was performed with the filterAndTrim command using the following settings to remove primers and low-quality sequences: truncLen = c(240, 200), truncQ = 2, trimLeft = c(25, 26), maxEE = c(2, 4). Paired reads with a minimum overlap of 100 bp were merged, and only sequences with 250–265 bp were kept for downstream analyses. Bacterial and archaeal taxonomy was assigned to the species level using the Silva 16S rRNA taxonomic database v.138.1 at the 80% confidence level (40, 41).

Fungal ITS reads were processed with the DADA2 workflow in a similar way, with a few modifications. Initially, sequence primers were removed using Cutadapt v.3.2 (42). The DADA2 function filterAndTrim command was used with the following parameters: truncLen = c(240, 200), maxN = 0, maxEE = c(2, 4), truncQ = 2, minLen = 50. Amplicon sequences were not size-selected due to the variable length of the ITS region. Fungal taxonomy was assigned using the UNITE ITS database v.8.2 (43), and a minimum bootstrap confidence of 80% was required for the assignment.

### Statistical analyses

Ecosystem functioning analyses were performed in R (R Core Team). We used a two-way analysis of variance (ANOVA) analysis to compare the impacts of microbial inoculum and generation on CO_2_ production and DOC abundance and estimated the percentage of variation that could be attributed to each significant term for the ANOVA (44). We assessed the range in functional variation and calculated the variability (coefficient of variation [CV]) in DOC and CO_2_ in each generation.

Microbiome composition analyses were performed in R using the “phyloseq” v.1.32.0 (45) and “vegan” v.2.5-7 (46) packages. Bacterial and fungal amplicon sequence variant (ASV) abundances were rarefied to the minimum sample size of 5,457 and 6,670 reads, respectively, and most samples approached saturation. Rarefied abundance data were used to calculate alpha diversity metrics (observed taxa, Shannon diversity), beta diversity metrics (Bray–Curtis distance, beta-dispersion), and abundance: function Pearson’s correlations. Alpha diversity measurements were compared using ANOVA and Tukey’s *post hoc* statistical tests. Beta diversity differences were compared with a Permutational multivariate analysis of variance test using the *adonis* function *in* vegan (46), pairwise_adonis package, and beta-dispersion was compared using ANOVA and Tukey’s tests. Bray–Curtis composition dissimilarities were correlated with DOC or CO_2_ differences for all pairwise sample combinations using Mantel tests. Briefly, sample pairwise distance matrices were constructed using the “dist” (“vegan” v.2.5-7), and the “mantel” function (“ecodist” v.2.0.7) was used with 999 permutations to compute correlations between compositional dissimilarity and DOC*CO_2_ dissimilarity for data subset by DOC stability. Pearson’s correlations between sample stability (DOC change from previous generation of serial propagation) and community composition (Bray–Curtis distance) change from previous generation for each individual community were also calculated. Since many Pearson’s correlations were calculated, the false discovery rate was controlled at 0.1 using the Benjamini–Hochberg procedure and adjusted *P*-values were calculated. Samples were categorized into two groups based on their DOC stability between generations. The absolute value of the difference in DOC between G_*n*_ and G_*n* − 1_ (ΔDOC) was calculated and used to assign samples in G_*n*_ to “most stable” or “least stable” groups. The median ΔDOC for all communities was 0.56 mg per g of DOC. Communities assigned to the most stable group were those with a ΔDOC in the 0–25th percentile (0 ≤ ΔDOC ≤ 0.28 mg DOC g^−1^), while communities assigned to the least stable group had a ΔDOC in the 75th–100th percentile (1.12 ≤ ΔDOC ≤ 3.92 mg DOC g^−1^).

### Microbial co-abundance networks

To examine how microbial interactions differed between the most and least functionally stable communities, taxa co-abundance networks were calculated using SparCC (47). Using rarefied data, bacterial ASV abundances were first grouped at the family level using the tax_glom function in Phyloseq (45) to reduce the total number of taxa used in the analysis (fungal abundances were analyzed at the ASV level). Then, samples were split into the most stable or least stable groups as described earlier. To calculate co-abundances of bacterial families or fungal ASVs, the python package SparCC3 (47) was run using the standard workflow, except that a custom script (Supplemental File 1) was used to calculate correlation thresholds using randomly shuffled abundance tables without replacement. Pseudo *P*-values were calculated using 10,000 bootstraps, and a *P*-value significance cutoff of 0.01 was used to identify significant co-occurrences. Cutoffs were determined independently for each network and represent equal levels of confidence in edges to account for differences in the distributions of relative abundances between groups. Networks were visualized using Cytoscape (v.3.9.1).

### Feature identification with RFINN machine learning

The machine learning tool RFINN (48) was used to identify microbial community features associated with high or low DOC abundance in all of the samples, rather than those subsets by functional stability. Random forest and neural network models were first trained to a randomly selected subset (80%) of the microcosm sample data to establish mathematical relationships between the rarefied taxa abundance (features) and ecosystem variables (DOC, CO_2_, and DOC Trajectory). Model efficiency and function predictability were assessed using the remaining 20% of data not included in the model-training step to compare predicted and measured values and calculate Pearson’s correlation coefficient (R) (48). Indicator species analysis identified taxa with significant (*P* < 0.05) associations with ecosystem functions, and these results were compared to model outputs.

## RESULTS

### Drivers of stable versus variable ecosystem functioning

**Fig 1:**
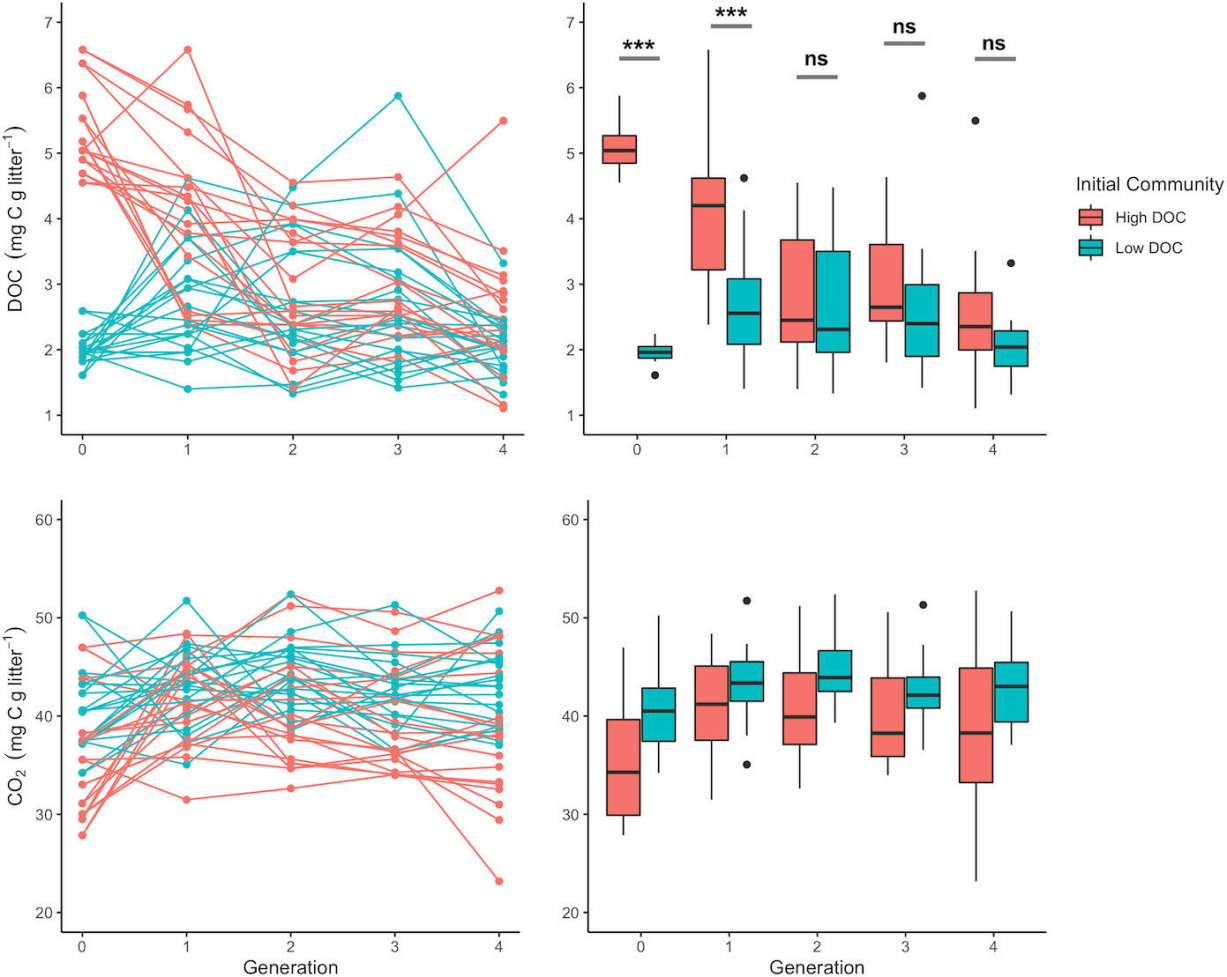
Measurements of total DOC and CO_2_ accumulation during all five generations of microcosms. Microcosms in the initial high and low DOC cohorts eventually converged in DOC accumulation, but DOC stability between generations varied greatly between microcosms. For DOC, two-way ANOVA showed significant Generation (*F* = 37.15, *P* < 0.001, degrees of freedom [df] = 4), Initial Community (*F* = 10.54, *P* < 0.001, df = 1), and Generation by Initial Community interaction (*F* = 9.91, *P* < 0.001, df = 4). Since there was a significant Generation by Initial Community interaction for DOC, significance bars indicate pairwise statistical differences (Tukey’s honestly significant difference) between high and low DOC Initial Community cohorts for each generation in the DOC boxplot, *** indicates *P* < 0.001, ns = not significantly different (*P* > 0.05). For CO_2_, two-way ANOVA showed significant Initial Community (*F* = 19.57, *P* < 0.001, df = 1) and Generation (*F* = 2.65, *P* = 0.035, df = 4) main effects only.

Following four generations of serial propagations of litter-decomposing communities, DOC abundance changed substantially between generations for some communities, while in others, DOC abundance remained more stable (Fig. 1; Fig. S2). DOC abundance across all microcosms decreased from G_0_ to G_4_ (median G_0_ 3.6 ± 1.8 to median G_4_ 2.1 ± 0.8 mg DOC g^−1^ litter [Fig. 1; Fig. S3]). However, the range between the minimum and maximum DOC abundance across all individual communities remained relatively consistent across generations (5.0, 5.2, 3.2, 4.5, 4.4 mg DOC g^−1^ litter). The variability in DOC abundance was greatest within G_0_ (CV = 0.48) and then decreased but remained constant across subsequent generations (CV = 0.36, 0.34, 0.34, 0.33). CO_2_ production varied twofold across all microcosms, and the overall range of CO_2_ production did not change from G_0_ to G_4_ (Fig. 1B; Fig. S3). Across generations, CO_2_ production was more stable than DOC abundance and remained consistent (G_0_ median = 37.5 mg CO_2_ g^−1^ litter, G_4_ median = 40.1 mg CO_2_ g^−1^ litter; CV = 0.16, 0.10, 0.11, 0.11, 0.15). For bacterial communities, compositional change between generations was positively correlated with the magnitude of change in DOC abundance (stability), but there was no significant correlation for fungal communities (Table 1A). Functional stability was negatively correlated with bacterial richness, but not fungal diversity (Table 1B and C), and compositional change was negatively correlated with bacterial richness and positively correlated with all three fungal diversity metrics (Table 1D and E).

**TABLE 1:** Pearson’s correlations of metadata associated with individual samples^*a*^

	Correlation	Pearson’s R	*P*-value (adjusted)
**A**	**∆Composition (bacterial) ~ functional stability (∆DOC**)	**0.259**	**<0.001**
	∆Composition (fungal) ~ functional stability (∆DOC)	0.010	0.34
**B**	**Functional stability (∆DOC) ~ bacterial richness**	**−0.196**	**0.04**
	Functional stability (∆DOC) ~ bacterial Shannon diversity	−0.144	0.16
	Functional stability (∆DOC) ~ bacterial evenness	−0.046	0.62
**C**	Functional stability (∆DOC) ~ fungal richness	0.047	0.65
	Functional stability (∆DOC) ~ fungal Shannon diversity	0.079	0.46
	Functional stability (∆DOC) ~ fungal evenness	0.054	0.64
**D**	**∆Composition (bacterial) ~ bacterial richness**	**−0.326**	**<0.001**
	∆Composition (bacterial) ~ bacterial Shannon diversity	−0.148	0.16
	∆Composition (bacterial) ~ Bacterial evenness	0.019	0.83
**E**	**∆Composition (fungal) ~ fungal richness**	**0.447**	**<0.001**
	**∆Composition (fungal) ~ fungal Shannon diversity**	**0.397**	**<0.001**
	**∆Composition (fungal) ~ fungal evenness**	**0.348**	**<0.001**

^*a*^ ∆Composition for each individual community in Gn represents the calculated Bray–Curtis dissimilarity between Gn and Gn − 1 for a given serially propagated community. Similarly, functional stability (∆DOC) for a sample in Gn was calculated as the difference in measured DOC values for a given propagated community between Gn and Gn − 1. Metadata for all samples across all generations were correlated. Significant correlations (Padj < 0.05) are highlighted in bold.

**Fig 2:**
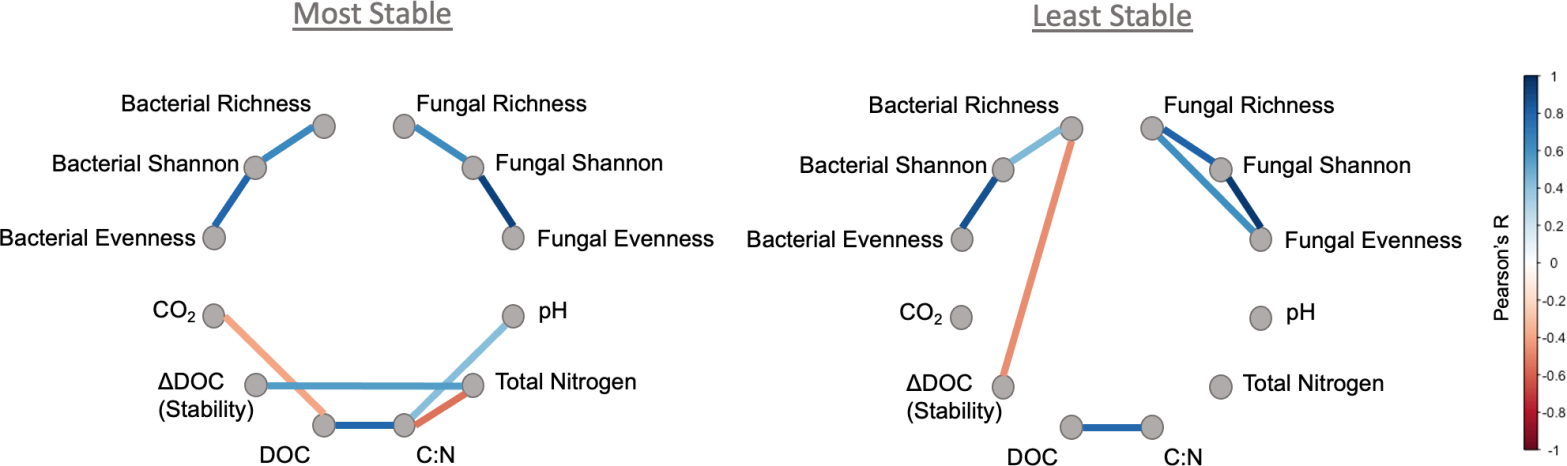
Pearson’s correlation networks of community diversity and microcosm measurements show distinct relationships among features occurred between the two community cohorts with the most stable and least stable DOC functions. Note that larger values of ΔDOC (stability) are less stable overall. The color of the line indicates the strength of the correlation coefficient R, and only significant correlations (*P*_adj_ < 0.05) are displayed. Refer to Table S3A and B for the complete results of this analysis in tabular form.

To better understand the drivers of functional stability across generations, we split the samples into two groups (most stable or least stable DOC; see Materials and Methods) and assessed correlations between community traits, microbial taxa, and ecosystem function within each group. Bacterial community compositional dissimilarity across all pairwise combinations of samples was significantly correlated with the pairwise dissimilarity of DOC abundance for the most stable group but not the ‘least stable’ group, nor to CO_2_ in either group (Table 2). Fungal community compositional dissimilarity and DOC dissimilarity were also only correlated in the most stable group (Table 2) but also correlated with CO_2_ dissimilarity in both the most and least stable groups. Despite links between community composition and DOC in the most stable samples, bacterial and fungal diversity metrics were not correlated with ΔDOC (functional stability) or other measurements in the most stable samples (Fig. 2). DOC abundance had a negative relationship with CO_2_ accumulation, and ΔDOC was positively correlated with TN in the most stable communities. In contrast, for the least stable group, bacterial richness was negatively correlated with ΔDOC, and DOC abundance was no longer correlated with CO_2_ in the least stable group of samples (Fig. 2). In both stability groups, bacterial and diversity metrics were not directly correlated with each other.

**TABLE 2:** Mantel correlation tests comparing community composition differences (pairwise Bray–Curtis dissimilarity) with pairwise differences in DOC or CO_2_ accumulation for all possible pairs of samples^*a*^

Bacteria	Most stable	Least stable
Bacteria		
Composition ~ DOC	***R = 0.254*** ***P = 0.010***	R = 0.018 *P* = 0.383
Composition ~ CO_2_	R = −0.024 *P* = 0.574	R = 0.018 *P* = 0.383
Composition ~ DOC*CO_2_	***R = 0.254*** ***P = 0.010***	R = 0.019 *P* = 0.367
Fungi		
Composition ~ DOC	***R = 0.219*** ***P = 0.001***	R = 0.076 *P* = 0.105
Composition ~ CO_2_	***R = 0.164*** ***P = 0.002***	***R = 0.149*** ***P = 0.007***
Composition ~ DOC*CO_2_	***R = 0.211*** ***P = 0.001***	R = 0.057 *P* = 0.172

^*a*^ Correlation coefficients (R) and *P*-values are displayed in the table, with significant Mantel correlations (*P* < 0.05) printed in bold and italics.

**Fig 3:**
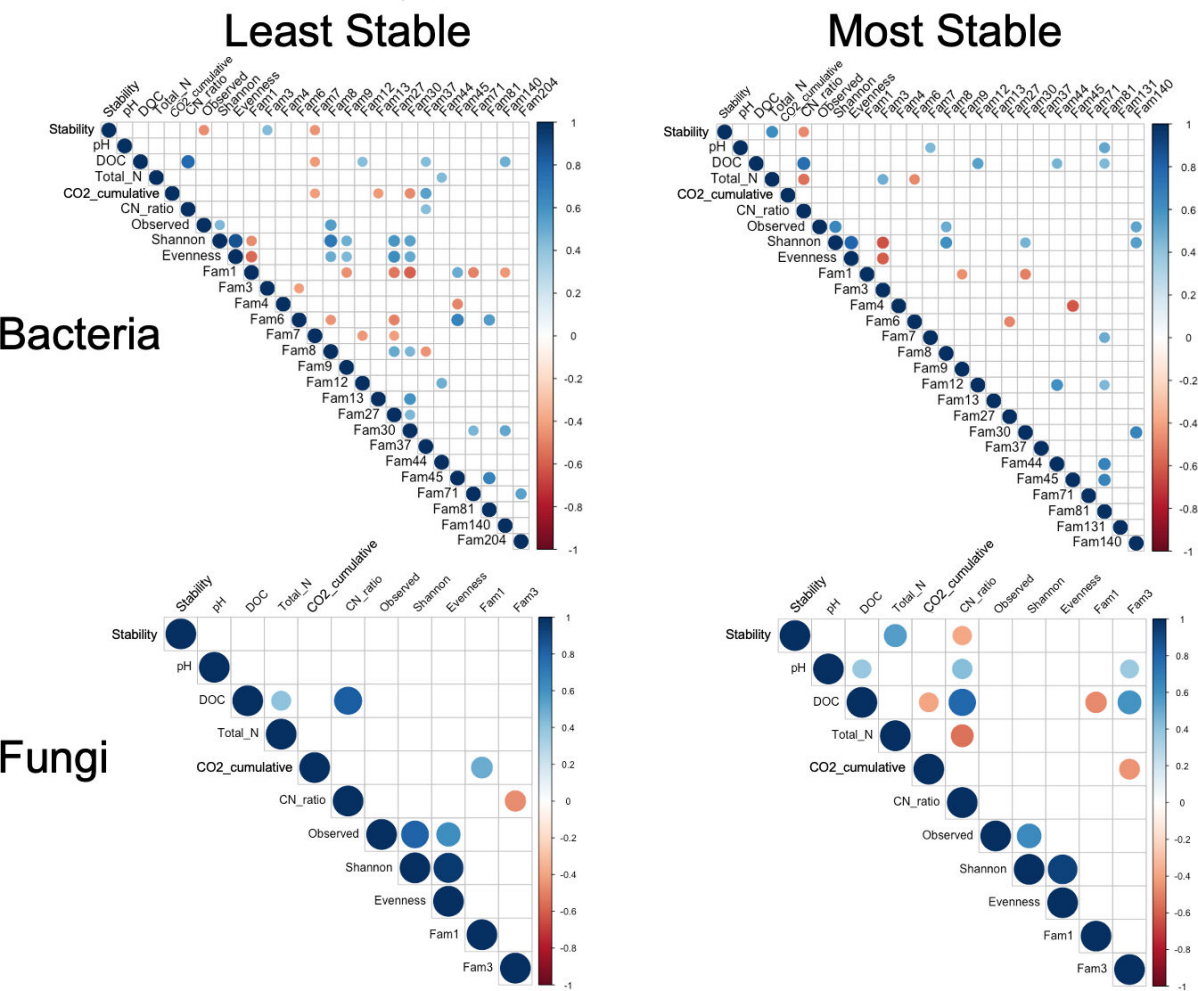
Pearson’s correlations between bulk measurements and abundances of bacterial or fungal families occurring in >80% of samples within each DOC stability cohort. Size and shading of dots in the matrices represent the Pearson’s R coefficient and are only displayed for correlations with *P*_adj_ < 0.05. Tabular versions displaying R and *P* values for these matrices are available in Table S3C through D. Note that “Stability” is the ΔDOC for a community between generations. All bacterial and fungal families listed here were present in both DOC groups, are notated as ‘Fam##’, and the names of these taxa are indexed as follows: **Bacterial taxa**: Pseudomonadaceae (1); Xanthomonadaceae (3); Alcaligenaceae (4); Chitinophagaceae (6); Rhizobiaceae (7); Devosiaceae (8); Flavobacteriaceae (9); Microbacteriaceae (12); Comamonadaceae (13); Sphingobacteriaceae (27); Caulobacteraceae (30); Xanthobacteraceae (37); Paenibacillaceae (44); Beijerinckiaceae (45); Oxalobacteraceae (71); Nocardiaceae (81); Micrococcaceae (131); Sphingomonadaceae (140); Rubritaleaceae (204). Fungal taxa: Chaetomiaceae (1); Bionectriaceae (3).

We searched for microbial features that explained DOC abundance and stability over multiple generations by performing Pearson’s correlations between taxa abundances, community traits, and functional measurements. Abundances of several microbial families were correlated with DOC abundance (Fig. 3). Among bacterial families, Microbacteriaceae was positively correlated with DOC abundance in both stability groups. Some taxa were only correlated with DOC abundance in one stability group. In the most stable group, Nocardiaceae and Paenibacillaceae (bacteria) were positively correlated with DOC. In the least stable group, the bacterial families, Xanthobacteriaceae and Sphingomonadaceae, were positively correlated, and Rhizobiaceae was negatively correlated with DOC abundance. Interestingly, no bacterial taxa were correlated with stability (ΔDOC) in the most stable group, but Xanthomonadaceae abundance was positively correlated with stability, and Rhizobiaceae was negatively correlated with stability in the least stable group. For fungi, Chaetomiaceae and Bionectriaceae abundances were negatively and positively correlated with DOC, respectively, in the most stable group only. Neither taxa correlated with stability in the least stable group.

**Fig 4:**
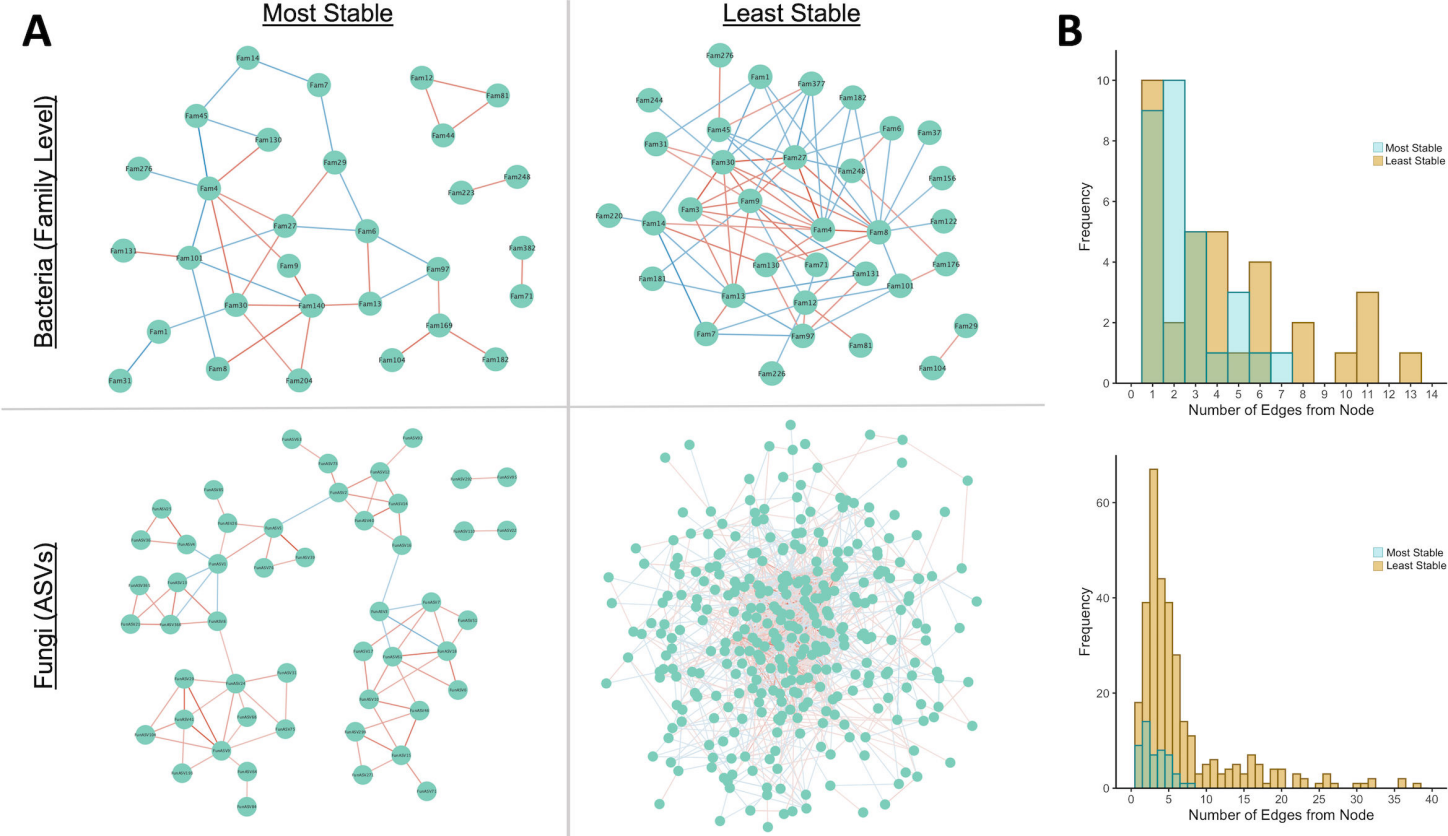
(A) Taxa co-abundance networks created using SparCC display qualitative differences in network structure, suggesting interaction patterns differed between the most and least stable communities. Bacterial co-abundance networks were calculated based on family level abundances to reduce the number of nodes, and fungal networks were calculated directly based on ASV abundances. Only significant edges (*P* < 0.01, 10,000 bootstraps) are displayed. Cutoffs were independently determined for each network, and edges represent equal levels of confidence while accounting for differences in taxa relative abundances between groups. Edges are colored and shaded according to the sign and weight of co-occurrence, respectively (blue = positive, red = negative relationship). (B) Histograms comparing the distributions of the number of edges connected to a single node.

Microbial co-abundance networks calculated with SparCC (47) showed different microbial interaction patterns between the most stable and least stable communities (Fig. 4). Networks were qualitatively distinct in their form and complexity between the two groups for both bacterial families and fungal ASVs (Fig. 4). Specifically, co-abundance networks were less complex and more fragmented in samples from the most stable group, relative to those of the least stable group. In the most stable group, few taxa co-occurred with many other taxa and multiple discrete interaction clusters were apparent, especially for the fungi. The least stable group was characterized by many taxa with links to many other taxa and less fragmentation (Fig. 4). Further, many taxa that were present in both stability groups were often connected to unique collections of taxa in each group.

### Changes in ecosystem functioning and microbial community composition across “generations”

Features associated with the temporal stability of ecosystem function prompted us to examine microbial trends across multiple generations. Overall, community re-assembly stabilized in later generations and legacy effects of microbiome origin were the largest drivers of diversity and compositional differences among microcosms. Microbial community origin, generation, and origin-by-generation interactions significantly influenced DOC abundance in all microcosms (ANOVA; origin: *F*_9,150_ = 32.2, *P* < 0.001, generation: *F*_4,150_ = 31.5, *P* < 0.001, and origin-by-generation: *F*_36,150_ = 6.6, *P* < 0.001). Microbiome origin explained about 33% of the estimated variance in DOC abundance, while generation contributed to 16% of the estimated variance (Fig. S4). DOC abundance was significantly correlated between generations for individually propagated microcosms (Fig. S2).

As with DOC, microbiome origin, generation, and origin-by-generation interactions significantly impacted CO_2_ production (origin: *F*_9,150_ = 11.7, *P* < 0.001, generation: *F*_4,150_ = 8.8, *P* < 0.001, and origin-by-generation: *F*_36,150_ = 3.1, *P* < 0.001). Microbiome origin explained the most variability (24%) in CO_2_ production (Fig. S4). For propagations of individual communities, CO_2_ production was only significantly correlated between later generations, including G_2_ to G_3_ (*R* ^2^ = 0.76, *P* < 0.001) and G_3_ to G_4_ (*R* ^2^ = 0.76, *P* = 0.001) (Fig. S2).

**Fig 5:**
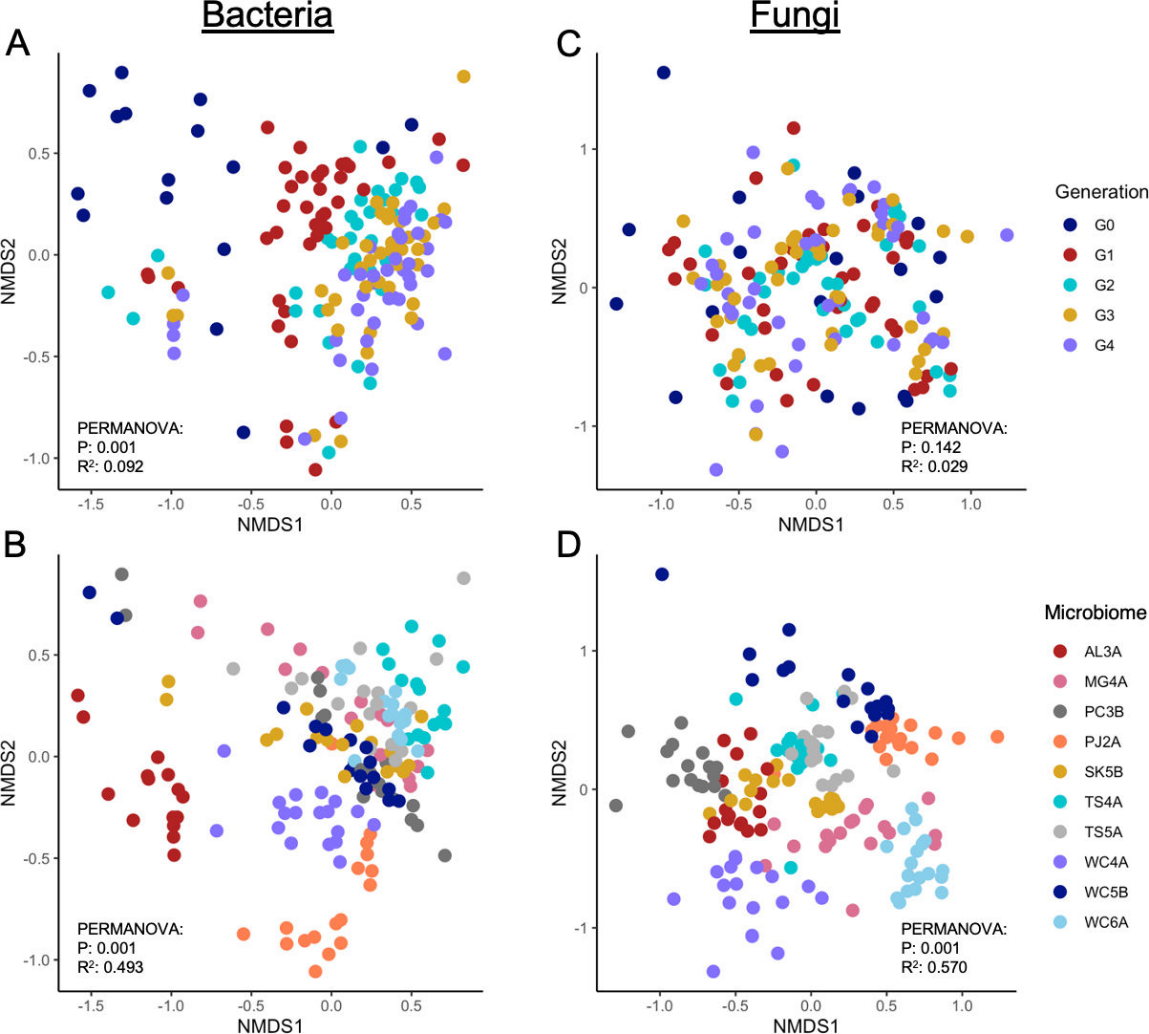
Non-metric multidimensional-scaled (NMDS) ordinations representing bacterial and fungal community composition. Bacterial community composition plotted according to generation (**A**) and soil origin (**B**). Fungal community composition plotted by generation (**C**) or soil origin (**D**). *Post hoc* analysis by pairwise *adonis* indicated all pairwise comparisons of composition were significant (*P*_adj_ < 0.05), except between the TS4A/TS5A fungal communities in (**D**).

Bacterial community composition was significantly impacted by microbiome origin (PERMANOVA, *R* ^2^ = 0.493, *P* = 0.001), generation (PERMANOVA, *R* ^2^ = 0.092, *P* = 0.001), and origin-by-generation interaction (PERMANOVA, *R* ^2^ = 0.182, *P* = 0.001) (Fig. 5A and B). All communities were distinct on the basis of microbiome origin (Fig. 5B). Bacterial community composition was significantly different between G_0_ and all later generations, G_1_ and G_3_, and G_1_ and G_4_ (pairwise *adonis* [Martinez Arbizu, 2020]) but was not significantly different between G_2_, G_3_, and G_4_, indicating communities initially changed rapidly and then stabilized (Fig. 5A). Fungal community composition was significantly impacted by microbiome origin (PERMANOVA, *R* ^2^ = 0.570, *P* = 0.001) and microbiome origin-by-generation interaction (PERMANOVA, *R* ^2^ = 0.120, *P* = 0.001) but not by generation alone (PERMANOVA, *R* ^2^ = 0.029, *P* = 0.14) (Fig. 5C and D).

**Fig 6:**
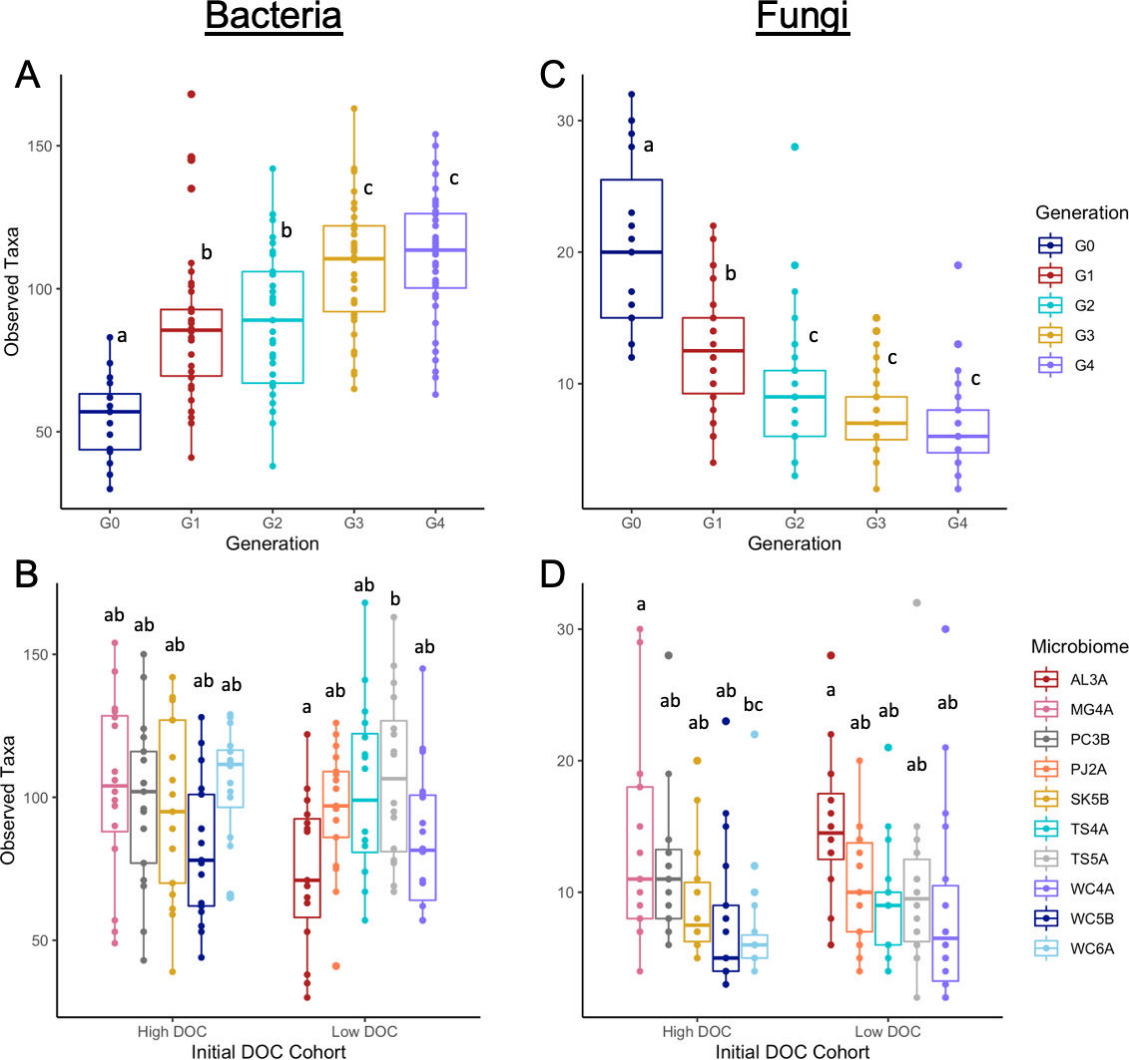
Bacterial and fungal alpha diversity. Bacterial richness by generation (**A**) or soil origin (**B**). Fungal richness by generation (**C**) or soil origin (**D**). ANOVA indicated significant (*P* < 0.05) generational and soil-origin effects on richness. Boxplots with unique letters indicate significant pairwise differences between microbiomes identified by Tukey’s honestly significant difference test. In plots B and D, microbiome sources are grouped by their initial high or low DOC cohorts, though these groups were not statistically different in species richness for bacteria or fungi.

Bacterial and fungal richness both varied by microbiome origin and showed opposite responses to serial propagation, with bacterial and fungal richness increasing and decreasing in subsequent generations, respectively (Fig. 6A through D). Despite being a closed system, these data suggest that some rare taxa may have been undetectable in the initial generation but proliferated in the microcosm environment. Shannon diversity and community evenness for both bacteria and fungi showed similar trends on richness (data not shown). Beta-dispersion was significantly different on the basis of microbiome origin but did not change across multiple generations for either bacterial or fungal communities (Fig. S5).

Indicator species analysis with the RFINN program (48) identified 42 and 16 bacterial ASVs as significant determinants of high and low DOC, respectively (Table S2). Fungi were not significant determinants of DOC. Predicted versus measured DOC abundances on test data showed high positive correlations (*R* ^2^ = 0.840) across all generations (data not shown), suggesting effective prediction of DOC based on community composition by the trained RFINN random-forest models.

## DISCUSSION

Our data show that differences in carbon flow driven by microbial origin can persist through successional processes in a common “soil-like” environment. Some communities showed greater stability of DOC abundance between generations compared to others, and we identified potential drivers of this phenomenon. Microbial community composition did not converge over time, but ecosystem function (DOC abundance and CO_2_ production) did, demonstrating compositional legacy effects and an increase in functional redundancy. Understanding the dynamics of microbial assembly and impacts on ecosystem function during repeated successional decomposition processes is central to manipulating and modeling soil C cycling.

### Drivers of functional stability

Microbial functional stability is an important factor in the elemental cycles of natural soils and is essential to microbiome engineering applications that aim to improve some aspect of microbial community performance (49). We posited that some microbial communities would exhibit greater relative stability in regard to their impacts on ecosystem functioning. Consistent with our hypothesis, DOC function was more stable between generations for some of the microcosm communities (Fig. 1). We split individual microcosms into two groups based on how much DOC abundance had changed from the previous generation (see Methods). Dividing the data in this way revealed distinct community and functional organization between the two stability groups that may help explain why microcosms behaved differently over the course of the experiment and allowed us to identify a variety of microbial traits at the community and individual taxa levels that were linked to greater functional stability.

**Fig 7:**
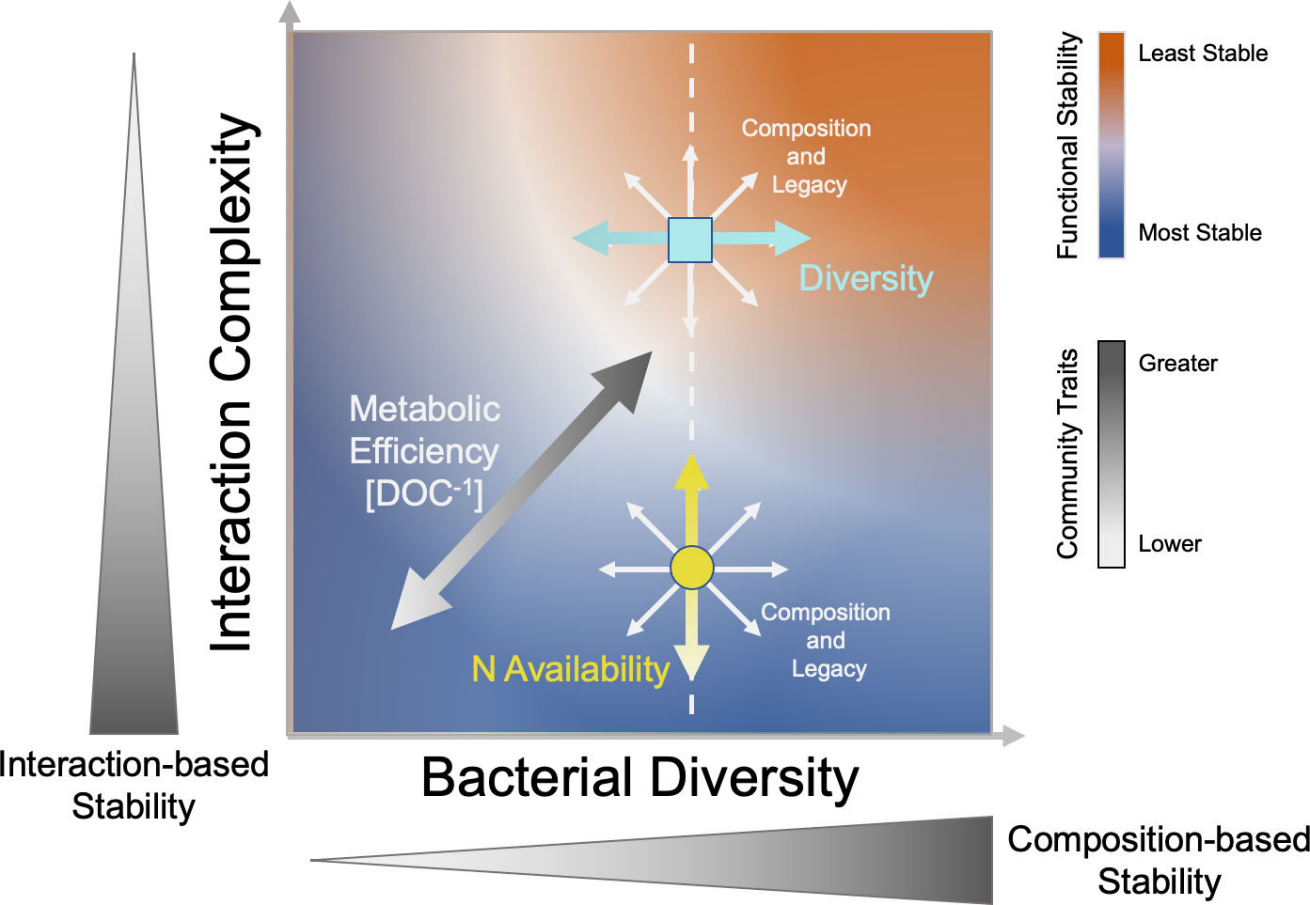
Conceptual plot summarizing how various microbial community traits may relate to functional stability. Functional stability (red to blue gradient) is linked to a tradeoff in interaction network complexity and bacterial community diversity. In general, lower interaction complexity and higher diversity are linked to greater interaction-based and composition-based stability, respectively (wedges along axes). The turquoise/square and yellow/circle points in the plot represent examples of the “most” and “least” stable communities in this study. In both, community composition and legacy effects can dictate where a community falls in this continuum (white arrows). For the “most stable” communities (yellow/circle), nitrogen availability may affect stability via interaction complexity (yellow arrows) since N was not correlated with diversity. Diversity may affect compositional and functional stability in the “least stable” communities in particular (turquoise arrows). A black to white gradient represents the range of possible values for interaction- and composition-based community stability components and metabolic efficiency (DOC concentration).

Our results highlight the potential importance of compositional stability, biotic interactions, and biotic–abiotic relationships in explaining the stability of DOC abundance during plant litter decomposition (Fig. 7). Given that abiotic parameters were controlled in the experimental design, observed variation in abiotic parameters between samples or stability groups is a consequence of functional differences between communities, while variations in biotic signatures may be either a cause and/or consequence of functional differences arising during community re-assembly. Across all samples, compositional stability was correlated with bacterial and fungal diversity metrics, but only bacterial richness and compositional stability were correlated with functional stability (ΔDOC) (Table 1 and Fig. 7). Given these relationships, when bacterial richness is high, composition and function would be expected to be more stable (lower ΔDOC and Δcomposition). In the most stable communities, Mantel correlations (Table 2) showed that community compositional differences scaled with functional differences between samples. Intuitively, this suggests that when DOC function was stable, compositionally similar communities were more likely to accumulate similar amounts of DOC and highlights composition as a potential driver of DOC abundance. In contrast, more similar communities were not likely to accumulate similar amounts of DOC in the least stable group, implying that additional factors may be decoupling composition and function. This is further supported by differences in correlation patterns between stability groups, which showed that interactions among biotic and environmental parameters were altered between the two stability groups (Fig. 2). The most stable group was characterized by no correlations between metrics of microbial diversity and DOC or other measurements. In terms of functional characteristics, the most stable communities were characterized by a clear trade-off between DOC abundance and respiration (CO_2_). Further, greater N availability was associated with functional destabilization (increase in ΔDOC) in the most stable communities. In the least stable group, bacterial richness interacted with functional stability (ΔDOC), but ΔDOC did not correlate with total N, nor did DOC and CO_2_ correlate. Noise in the data driven by inactive cells from eDNA-based community samples may explain why some of the reported community-level correlations were relatively weak; but importantly, stability may also be further explained by fine-scale community features and interactions that are less resolved by broad-scale community metrics. Multiple studies have suggested that microbial interactions can impact microbiome stability, where competition and decreased ecological interactions generally increase microbiome stability (50, 51). Shifts in composition and diversity can alter interaction networks, changing C and N utilization via modified cross-feeding and niche availability (52). Differences in fungal: bacterial community structure have also been linked to changing nitrogen availability and positively correlated with microbial metabolic efficiency (53), a likely driver of DOC abundance. Thus, the stability of DOC abundance and carbon flow could be biologically controlled through differential structuring of microbial community composition and activity via modified interaction networks relevant to carbon and nitrogen exchange. Observed changes at the community level suggest that interaction networks were modified between stability groups.

In agreement with the community-level analyses, taxa co-abundance analysis revealed striking differences in inferred interaction patterns between the most stable and least stable communities. Interaction networks were distinct in their structure and complexity between the most and least stable communities (Fig. 4). Specifically, the most stable communities had networks that were more fragmented and less complex, and taxa shared between the most and least stable community networks frequently interacted directly with different taxa. This distinction shows how differences in microbial interaction networks may explain variability in functional stability. Greater network complexity and increased interactions may allow small changes in taxa abundance or activity to have a rippling effect throughout the network, altering overall composition and destabilizing function as communities and diversity shift in response (54). A more fragmented network with fewer connections between taxa may help buffer against dramatic shifts in DOC function resulting from small changes in the abundances of taxa. Community compositional stability depends on a balance between richness and interaction connectivity (Fig. 7), where communities may be stable either when richness is high but connectivity is low or when richness is low but connectivity is high (54, 55). Given that species richness did not differ between the most and least stable communities (data not shown), if the number of interactions increases, the community will be more likely to be unstable (54, 55). Destabilization in highly connected networks can be mitigated by either segmentation of the interaction network or decreasing the strength of the interactions (55). These predictions are consistent with interaction networks observed for the two stability levels. In this experiment, bacterial richness was negatively associated with changes in bacterial composition, and compositional stability was positively correlated with functional stability in the microcosm system. Therefore, with a clear difference in interaction connectivity, our results strongly agree with interaction-based predictions of compositional (and functional) stability and show how changes in microbial interactions may alter diversity and composition, and consequentially, ecosystem function (Fig. 7).

Identifying taxa that are associated with functional stability or promote “high” or “low” DOC abundance is important for understanding microbial drivers of soil carbon storage (7). At the family level, six bacterial families and two fungal families were correlated with DOC abundance across both stability groups. Only one bacterial family (Microbacteriaceae) showed similar relationships in both groups, while the remaining taxa were uniquely correlated with DOC in only one stability group (Fig. 3). All of the taxa correlating with DOC were present in samples from both stability groups, thus stability-specific correlations were not a consequence of taxa being absent in one group. This implies that some taxa are only associated with DOC under certain contexts and is consistent with the co-abundance analysis results showing that microbial associations with other features changed between the unique contexts of each stability group. It is possible that some combination of these taxa directly stabilize or de-stabilize DOC function over time. Alternatively, other factors may control DOC stability and indirectly promote the activities of these taxa. Nevertheless, one approach to increase functional stability when optimizing communities to modify carbon flow may be to promote the growth of taxa that correlate with DOC abundance under the most stable conditions. Despite differences in the correlations of key taxa between the most stable and least stable groups, many of the taxa identified with correlation analysis were also found to be significant determinants of overall DOC abundance by the RFINN analysis (Table S2). For example, taxa positively correlating with DOC in either group closely matched to those identified as determinants of high DOC across all samples (i.e., Paenibacillaceae and Microbacteriaceae), and those negatively correlated with DOC (Rhizobiaceae) were also identified as determinants of low DOC by RFINN, although this depended on the specific sequence variants within the Rhizobiaceae family. Agreement between the two analysis approaches increases confidence that these taxa are key players in regulating both DOC stability and abundance during the early decomposition of blue grama plant litter. No fungi were associated with either high or low DOC by the RFINN analysis, contrary to the previous litter decomposition studies (7), but this may be a consequence of the serial community propagation method selection against some fungi.

### Community composition and ecosystem function over time

Microbial origin was key in shaping composition, and these legacy effects were stronger than the generation (temporal) effects in this experiment. Microbial community function converged to a predominately low DOC phenotype by the second generation, despite a nearly threefold difference in mean DOC abundance between the original high and low DOC cohorts during the initial generation (Fig. 1). Although bacterial community composition shifted substantially between the first few generations, composition was stabilized by the second generation, but microbial community composition of these cohorts did not converge in later generations (Fig. 5). The lack of compositional convergence suggests that legacy effects, microbial interactions, microbe–environment feedbacks, and/or stochastic effects were important drivers of composition in this system. This agrees with previous studies that have demonstrated strong compositional legacy effects and functional redundancy in succession experiments (30, 56 - 58). This result implies that functional convergence towards the low DOC phenotype may be a more energetically favorable and likely outcome following multiple rounds of serial propagation, and highlights the likelihood of alternative stable states in community composition and functioning. An alternative outcome could have been that composition also converged in later generations; however, this would likely only be expected under extremely harsh conditions with strong selective community filtering (59, 60). Our data show that community structure and interactions impacted both functional stability and the magnitude of DOC abundance, and that fragmentation of microbial interactions was associated with more stability in DOC abundance. This could lead to higher DOC abundance by altering a community’s ability to exploit available niches and resources by disconnecting pathways of metabolite exchange among taxa. Shifts in community composition following repeated assembly (propagation) events are likely to fill empty niches and utilize available resources eventually and may explain the observed functional convergence and why a low DOC phenotype may be favored. Functional convergence without compositional convergence demonstrates functional redundancy across multiple plant litter–degrading communities.

Microbiome origin and origin-by-generation interactions impacted community composition to a greater degree than generation alone for both bacterial and fungal communities, and the effects were strongest in fungal communities. Biological legacy effects have been shown to be major drivers of fungal composition at multi-year timescales during plant litter decomposition in a field experiment (58); however, the lack of temporal effects may also be a consequence of niche persistence, slow fungal growth rates during the short 28-day incubation, or failure of the liquid resuspension-inoculation method to effectively transfer some fungal taxa could lead to lower fungal richness and dispersion during later generations. In soils, abiotic effects on bacterial composition have been reported to be much larger than biotic origin signatures (60). In this experiment, the repetitive dilution approach used to inoculate the microcosms over multiple generations would have greatly reduced the carryover and influence of disparate abiotic factors from each natural soil inoculum, especially in later generations, leaving microbiome legacy to be a primary driver of community assembly. The addition of fresh blue grama litter in each new generation of microcosms likely provided favorable conditions with a relatively high resource supply, allowing many different types of microbes to thrive and explain why function, but not composition, converged over time. When source communities are preconditioned to their new habitat, community development is expected to be more reproducible (29, 30). Continuing the experiment for more generations may have altered composition and/or function over longer timeframes (58); however, higher compositional and functional stability in later generations contradicts this possibility, and in the absence of harsh environmental conditions that might select for the survival of specific organisms, compositional convergence would also not be expected (59, 60).

During plant litter decomposition, differences in community composition and diversity may be important factors that predict the stability and magnitude of microbial ecosystem functions (Fig. 7). Similarly, inoculum-driven differences in methane production have been shown to persist in bioreactors operated under similar conditions (57). In contrast, a multi-year study found that microbial origin significantly impacted plant litter decomposition rates during the first year of the experiment, but these effects diminished in subsequent years and microbial origin played a less important role than environment or litter type in the long term (58). The diminishing impacts of microbial origin as timescale increases may be due to the interaction of evolutionary and ecological timescales, such as the presence of a time lag in environmental selection (61); however, the duration of our study was less than 1 year, thus factors associated with longer timescales may not have been very influential in our plant litter decomposition system.

In the context of bioengineering, the origin of the microbial community, its overall composition, and its individual members have a significant potential to dictate the outcome of ecosystem function. Accounting for microbiome legacies that favor the formation of communities with stabilizing features and features associated with the desired function may promote the reliability of the programmed ecosystem functioning. In our experimental system, choosing an inoculum that favors succession leading to a less complex network of microbial interactions and high bacterial diversity is likely to minimize compositional and functional shifts over time (Fig. 7). Managing bacterial and fungal interactions through manipulations of nutrient availability and control of community structure may also facilitate functional stability and high DOC abundance in this system (Fig. 7). Although our experimental design utilized complex communities from many different soil sources, translating these findings to effectively manage microbial communities at a field scale still poses major challenges due to additional factors that we were able to control for in the microcosm system. Our experiment considered communities that were inoculated into a sterile environment where they did not encounter or interact with a resident community and were grown under stable, controlled environmental conditions. Applying our findings to inoculate natural soil communities with the aim of optimizing stable ecosystem functioning would likely show different outcomes. In future work, it will be necessary to better understand how microbial features linked to the magnitude and stability of DOC abundance (or other ecosystem functions) interact with factors that permit successful community coalescence in dynamic natural environments. Alternatively, management methods that attempt to directly optimize existing communities for features associated with stability and the desired function may provide another path forward to engineer communities, but this will also require further experimentation. Nevertheless, our findings provide some clarity on features of microbial communities that may promote or destabilize DOC abundance in complex plant litter–degrading communities, which may be useful for predicting functional stability and carbon cycling in natural soils and informing microbial engineering strategies to optimize desired ecosystem functions.

### Conclusion

Predictability of microbial community function over long timescales and repetitive community assembly events is essential to achieve a desired stable function. In this experiment, we assessed the stability and persistence of litter decomposer microbial community composition and function. Serial propagation of high and low DOC accumulating communities allowed us to repeatedly perturb and reassemble complex communities to understand how composition and function changed over multiple generations. We found that community composition and the resulting effects on interaction networks in microbial communities explained differences in the functional stability of plant litter–decomposing communities, and that legacy effects were also important in determining composition and function. Our results suggest that controlling composition and abiotic factors to limit microbial interactions could increase the stability and reliability of DOC abundance in plant litter–decomposing communities over the long term. Understanding these factors will provide clarity on mechanisms controlling carbon flow in natural soil environments, and potentially allow for the optimization of engineered communities designed to enhance carbon sequestration or perform other desired ecosystem functions.

## Data Availability

Raw sequence read data are publicly available in the NCBI Sequence Read Archive under accession PRJNA805211. Processed microbiome abundance, taxonomy, and metadata are provided as a supplemental file (Table S4A through C).

## SUPPLEMENTAL MATERIAL

FIG S1 msystems.01220-22-s0001.tif Experimental Design. 53 microbial communities were inoculated into microcosms and incubated for 28 days (G_0_). Communities from microcosms that produced the highest and lowest mean DOC were selected for serial propagation. Each selected microcosm was serially propagated in duplicate for G_1_, then each microcosm was directly propagated once in following generations.

FIG S2 msystems.01220-22-s0002.tif Pearson’s correlations between successive generations show that ecosystem function (DOC, or CO_2_ accumulation) tends to resemble that of the previous generation.

FIG S3 msystems.01220-22-s0003.tif Distribution of DOC and CO_2_ measurements by generation.

FIG S4 msystems.01220-22-s0004.tif Variance component analysis showing the contributions of soil origin and generation to the overall variance of DOC and CO_2_ measurements.

FIG S5 msystems.01220-22-s0005.tif Beta-dispersion analysis by generation or source microbiome.

SUPPLEMENTAL FILE 1 msystems.01220-22-s0006.txt Python script used for calculation of taxa-abundance networks with SparCC3.

TABLE S1 msystems.01220-22-s0007.docx Sampling locations of the natural soil communities chosen for propagation following the initial generation of microcosms

TABLE S2 msystems.01220-22-s0008.docx RFINN prediction of DOC function with random forest and neural-network modeling. Bacterial ASVs significantly (P < 0.05) associated with high or low DOC accumulation identified through indicator species analysis. RFINN did not identify fungi as major predictors of DOC function and are not included in the table. ASVs matching to bacterial families that significantly correlated with DOC in the ‘most stable’ and ‘least stable’ groups (Fig. 3) are shown in bold and marked with a star (*).

TABLE S3 msystems.01220-22-s0009.xlsx
**A**) Pearson correlation results for data displayed in Fig. 2. This table displays results for correlations calculated for the “Most Stable’ communities. Significant correlations (P < 0.05) are shaded. **B**) Pearson correlation results for data displayed in Fig. 2. This table displays results for correlations calculated for the “Least Stable’ communities. Significant correlations (P < 0.05) are shaded. **C**) Bacterial Families:Bulk Measurement Pearson's correlations for the "Most Stable" cohort of samples (Fig. 3). **D**) Bacterial Families:Bulk Measurement Pearson's correlations for the "Least Stable" cohort of samples (Fig. 3). **E**) Fungal Families:Bulk Measurement Pearson's correlations for the "Most Stable" cohort of samples (Fig. 3). **F**) Fungal Families:Bulk Measurement Pearson's correlations for the "Least Stable" cohort of samples (Fig. 3)

TABLE S4 msystems.01220-22-s0010.xlsx A) 16S (Bacterial) combined abundance and taxonomy table. B) ITS (Fungal) combined abundance and taxonomy table. C) Experimental Metadata

ASM does not own the copyrights to Supplemental Material that may be linked to, or accessed through, an article. The authors have granted ASM a non-exclusive, world-wide license to publish the Supplemental Material files. Please contact the corresponding author directly for reuse.
