# Supplementary figures and images for: Drivers of stability and transience in composition-functioning links during serial propagation of litter-decomposing microbial communities
Source: mSystems. 2023 May 3;8(3):e01220-22. doi: 10.1128/msystems.01220-22 (PMC10308953; doi:10.1128/msystems.01220-22)

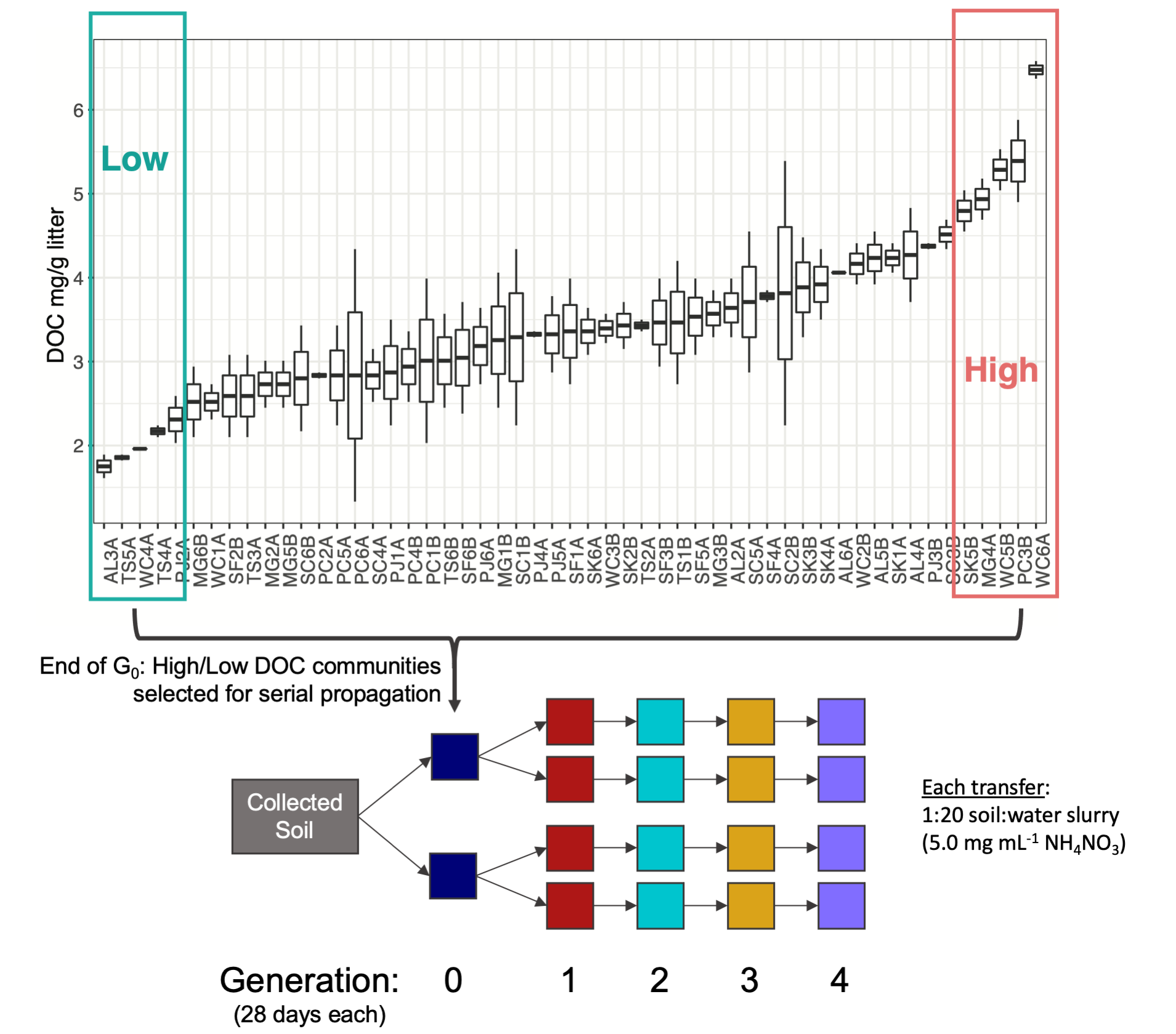

Supplement: FIG S1 — Experimental Design. 53 microbial communities were inoculated into microcosms and incubated for 28 days (G0). Communities from microcosms that produced the highest and lowest mean DOC were selected for serial propagation. Each selected microcosm was serially propagated in duplicate for G1, then each microcosm was directly propagated once in following generations. [file msystems.01220-22-s0001.tif]

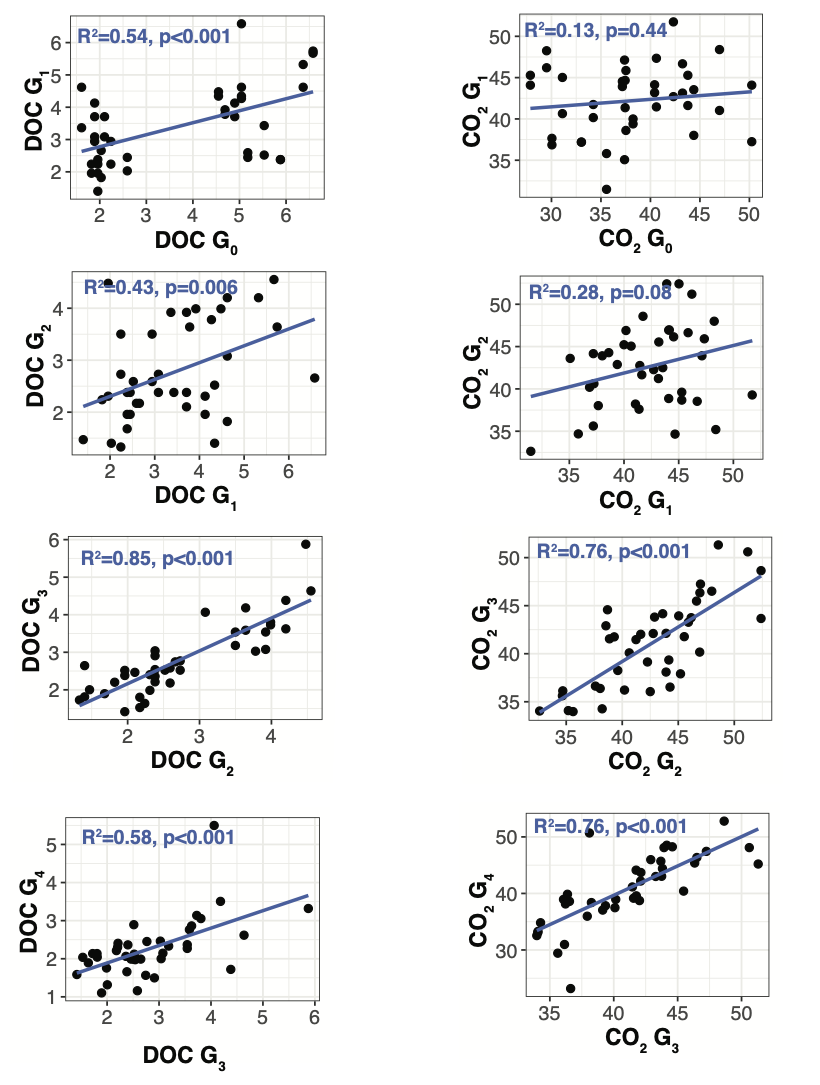

Supplement: FIG S2 — Pearson’s correlations between successive generations show that ecosystem function (DOC, or CO2 accumulation) tends to resemble that of the previous generation. [file msystems.01220-22-s0002.tif]

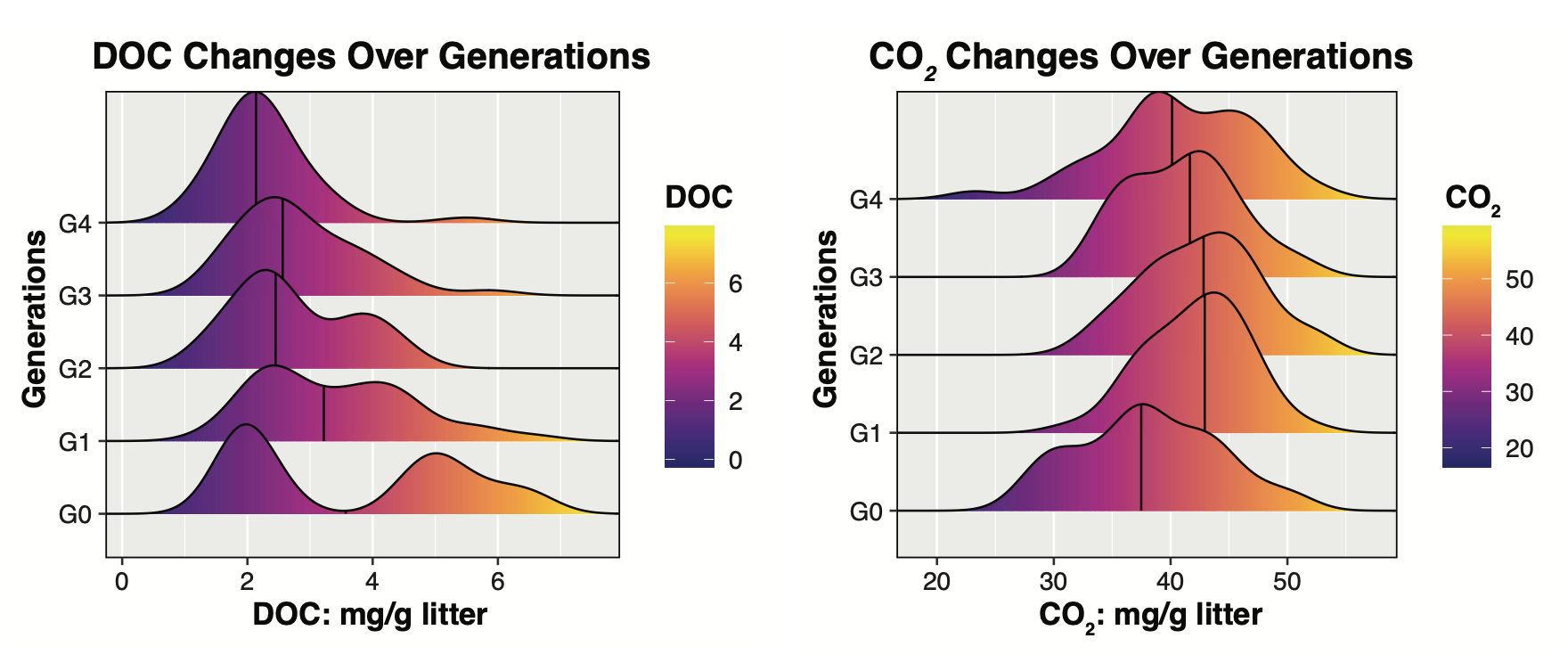

Supplement: FIG S3 — Distribution of DOC and CO2 measurements by generation. [file msystems.01220-22-s0003.tif]

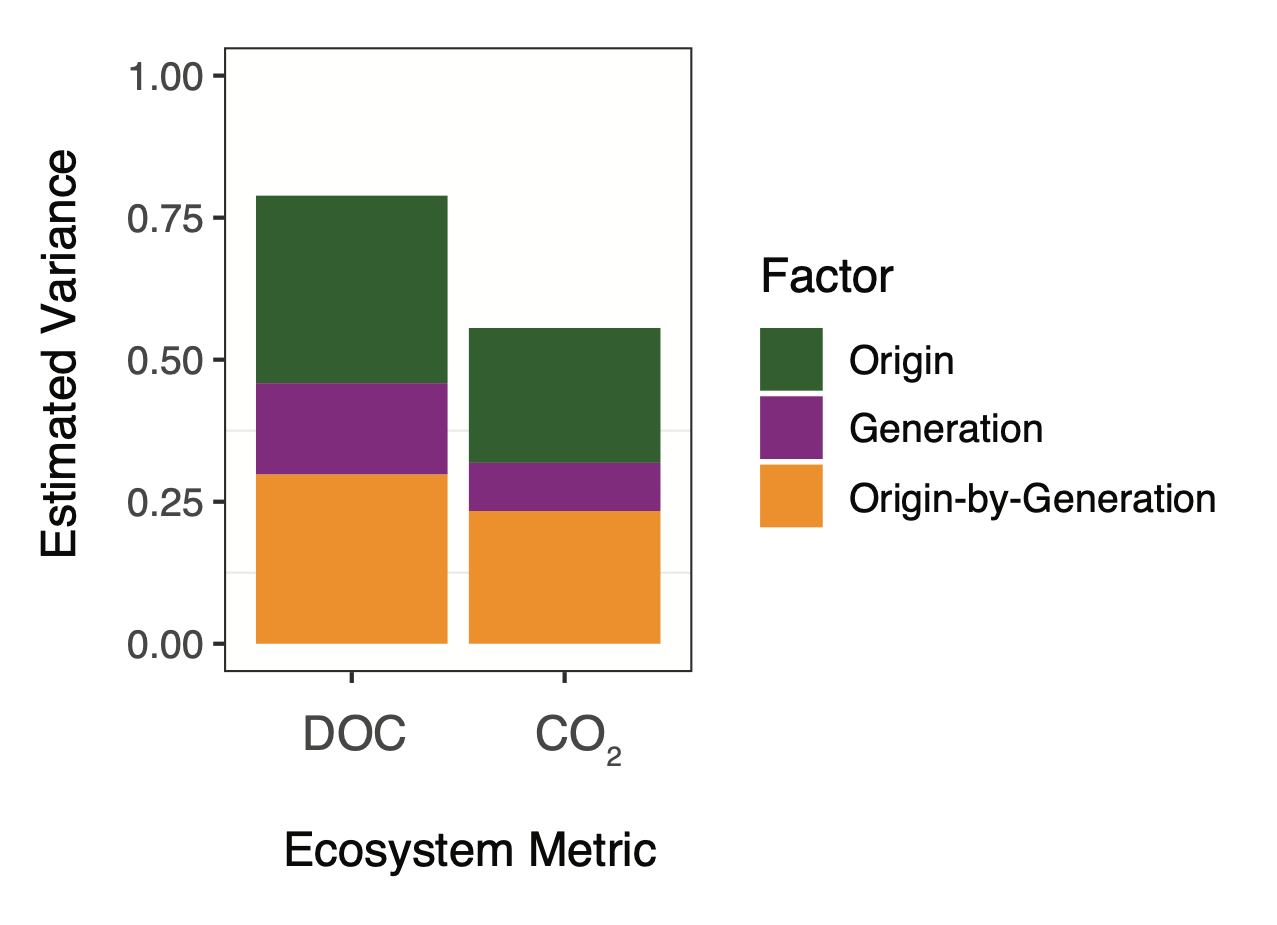

Supplement: FIG S4 — Variance component analysis showing the contributions of soil origin and generation to the overall variance of DOC and CO2 measurements. [file msystems.01220-22-s0004.tif]

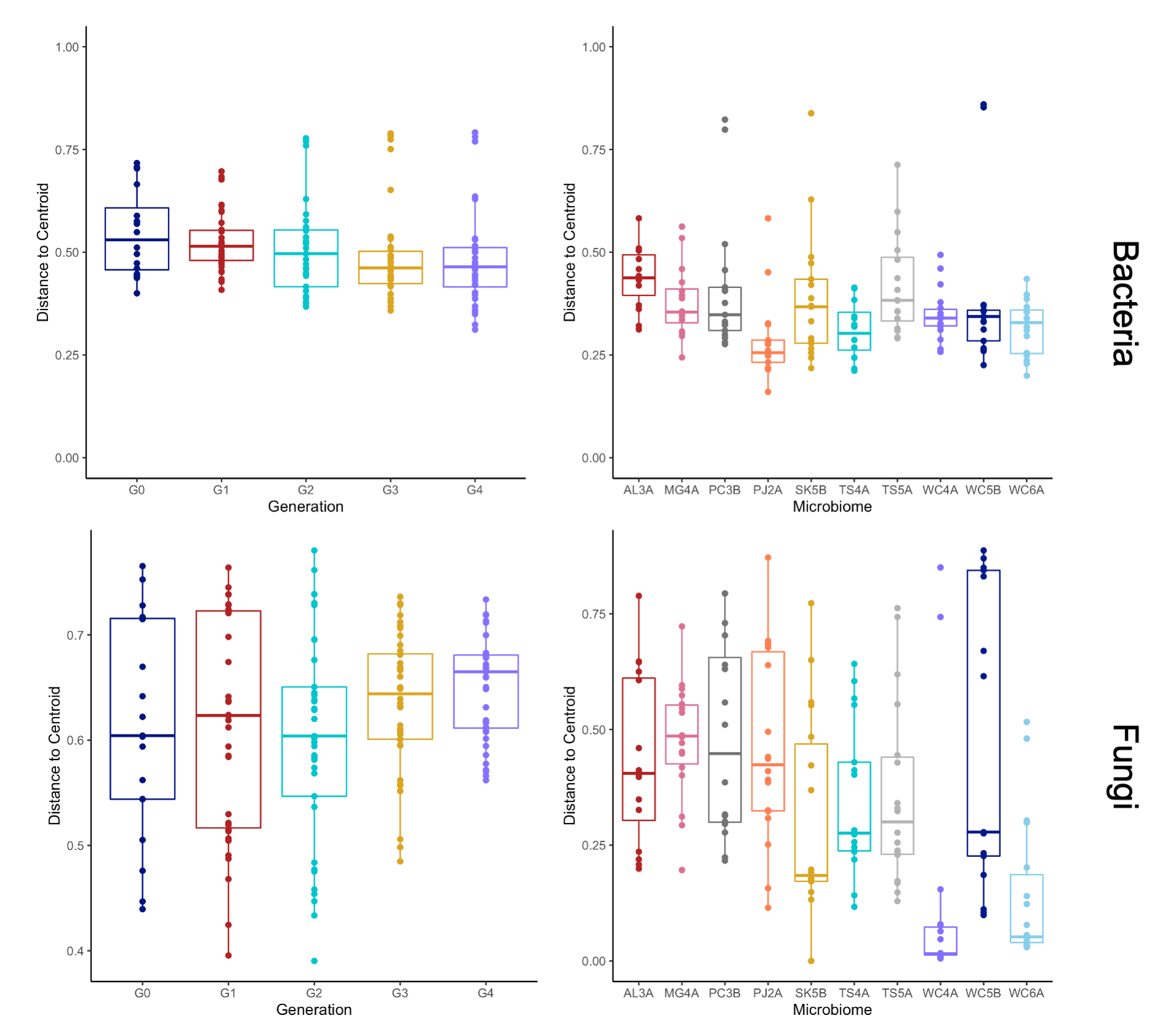

Supplement: FIG S5 — Beta-dispersion analysis by generation or source microbiome. [file msystems.01220-22-s0005.tif]
