# Supplementary material for: Drivers of stability and transience in composition-functioning links during serial propagation of litter-decomposing microbial communities
Source: mSystems. 2023 May 3;8(3):e01220-22. doi: 10.1128/msystems.01220-22 (PMC10308953; doi:10.1128/msystems.01220-22)
Supplement: TABLE S1 — Sampling locations of the natural soil communities chosen for propagation following the initial generation of microcosms [file msystems.01220-22-s0007.docx]

| Sample | Site | Location | Elevation (m) | Collection Date |
| --- | --- | --- | --- | --- |
| AL3A | South Fork, CO | 37.595° N, 106.727° W | 2593 | Oct. 15 2019 |
| MG4A | Magdalena Ridge, NM | 33.996° N, 107.151° W | 2613 | Oct. 23 2019 |
| PC3B | Pecos, NM | 35.825° N, 105.657° W | 2530 | Oct. 18 2019 |
| PJ2A | Pajarito Mountain, NM | 35.876° N, 106.344° W | 2349 | Oct. 9 2019 |
| SK5B | San Francisco Peaks, AZ | 35.283° N, 111.711° W | 2320 | Oct. 13 2019 |
| TS4A | Taos Ski Valley, NM | 36.596° N, 105.475° W | 2779 | Oct. 21 2019 |
| TS5A | Taos Ski Valley, NM | 36.597° N, 105.449° W | 2903 | Oct. 21 2019 |
| WC4A | Wolf Creek Pass, CO | 37.451° N, 106.888° W | 2660 | Oct. 15 2019 |
| WC5B | Wolf Creek Pass, CO | 37.473° N, 106.870° W | 2867 | Oct. 15 2019 |
| WC6A | Wolf Creek Pass, CO | 37.485° N, 106.836° W | 3081 | Oct. 15 2019 |

Table S1: Sampling locations of the natural soil communities chosen for propagation following the initial generation of microcosms.
