# Supplementary material for: Drivers of stability and transience in composition-functioning links during serial propagation of litter-decomposing microbial communities
Source: mSystems. 2023 May 3;8(3):e01220-22. doi: 10.1128/msystems.01220-22 (PMC10308953; doi:10.1128/msystems.01220-22)
Supplement: TABLE S2 — RFINN prediction of DOC function with random forest and neural-network modeling. Bacterial ASVs significantly (P < 0.05) associated with high or low DOC accumulation identified through indicator species analysis. RFINN did not identify fungi as major predictors of DOC function and are not included in the table. ASVs matching to bacterial families that significantly correlated with DOC in the ‘most stable’ and ‘least stable’ groups (Fig. 3) are shown in bold and marked with a star (*). [file msystems.01220-22-s0008.docx]

| **High DOC** | **Low DOC** |
| --- | --- |
| ASV 45 (Beijerinckiaceae) | ASV 90 (Burkholderiaceae) |
| ASV 166 (Beijerinckiaceae) | ASV 6 (Chitinophagaceae) |
| ASV 300 (Beijerinckiaceae) | ASV 49 (Devosiaceae) |
| ASV 255 (Brevibacillaceae) | ASV 165 (Oxalobacteraceae) |
| ASV 30 (Caulobacteraceae) | ASV 379 (Pirellulaceae) |
| ASV 250 (Caulobacteraceae) | ASV 22 (Pseudomonadaceae) |
| ASV 43 (Comamonadaceae) | ASV 23 (Pseudomonadaceae) |
| ASV 39 (Devosiaceae) | ASV 33 (Pseudomonadaceae) |
| ASV 65 (Devosiaceae) | ***ASV 61 (Rhizobiaceae)** |
| ASV 153 (Devosiaceae) | ***ASV 75 (Rhizobiaceae)** |
| ASV 32 (Flavobacteriaceae) | ASV 28 (Sphingobacteriaceae) |
| ASV 132 (Flavobacteriaceae) | ASV 181 (Steroidobacteraceae) |
| ASV 144 (Flavobacteriaceae) | ***ASV 156 (Xanthobacteraceae)** |
| ASV 175 (Hyphomicrobiaceae) | ASV 3 (Xanthomonadaceae) |
| ***ASV 12 (Microbacteriaceae)** | ASV 15 (Unknown Family) |
| ***ASV 143 (Microbacteriaceae)** | ASV 111 (Unknown Family) |
| ***ASV 152 (Nocardiaceae)** |  |
| ***ASV 44 (Paenibacillaceae)** |  |
| ***ASV 105 (Paenibacillaceae)** |  |
| ***ASV 108 (Paenibacillaceae)** |  |
| ***ASV 201 (Paenibacillaceae)** |  |
| ***ASV 254 (Paenibacillaceae)** |  |
| ***ASV 289 (Paenibacillaceae)** |  |
| ***ASV 304 (Paenibacillaceae)** |  |
| ***ASV 338 (Paenibacillaceae)** |  |
| ***ASV 400 (Paenibacillaceae)** |  |
| ASV 357 (Planococcaceae) |  |
| ***ASV 113 (Rhizobiaceae)** |  |
| ***ASV 160 (Rhizobiaceae)** |  |
| ***ASV 225 (Rhizobiaceae)** |  |
| ***ASV 299 (Rhizobiaceae)** |  |
| ***ASV 437 (Rhizobiaceae)** |  |
| ASV 579 (Solirubrobacteraceae) |  |
| ASV 54 (Sphingobacteriaceae) |  |
| ASV 125 (Sphingobacteriaceae) |  |
| ***ASV 139 (Sphingomonadaceae)** |  |
| ***ASV 294 (Sphingomonadaceae)** |  |
| ***ASV 37 (Xanthobacteraceae)** |  |
| ASV 102 (Xanthomonadaceae) |  |
| ASV 172 (Xanthomonadaceae) |  |
| ASV 177 (Xanthomonadaceae) |  |
| ASV 29 (Weeksellaceae) |  |

Table S2: RFINN prediction of DOC function with random forest and neural-network modeling. Bacterial ASVs significantly (P < 0.05) associated with high or low DOC accumulation identified through indicator species analysis. RFINN did not identify fungi as major predictors of DOC function and are not included in the table. ASVs matching to bacterial families that significantly correlated with DOC in the ‘most stable’ and ‘least stable’ groups (Fig. 3) are shown in bold and marked with a star (*).
